# IDEAL: A Community–Academic–Governmental Collaboration Toward Improving Evidence-Based Data Collection on Race and Ethnicity

**DOI:** 10.5888/pcd20.230029

**Published:** 2023-10-12

**Authors:** Farah Kader, Lan N. Ðoàn, Matthew K. Chin, Maya Scherer, Luisa Cárdenas, Lloyd Feng, Vanessa Leung, Anita Gundanna, Matthew Lee, Rienna Russo, Olugbenga G. Ogedegbe, Iyanrick John, Ilseung Cho, Simona C. Kwon, Stella S. Yi

**Affiliations:** 1 Department of Population Health, New York University Grossman School of Medicine, New York, New York; 2 Center for Evaluation and Applied Research, The New York Academy of Medicine, New York, New York; 3 Coalition for Asian American Children and Families, New York, New York; 4 ChangeLab Solutions, Oakland, California; 5 Department of Medicine, New York University Grossman School of Medicine, New York, New York

Equity in health data requires careful consideration of the collection, analysis, interpretation, and communication practices of data on race and ethnicity and meaningful inclusion of populations that datasets represent. From a racial equity perspective, each step of the data life cycle must center on community engagement and shared decision-making. Upon executing a data equity framework, datasets may become more complete, accurate representations of racially and ethnically heterogeneous communities and drive data-driven policy decisions to advance equity (1,2).

However, outdated data collection practices present obstacles to effectively responding to public health crises and eliminating racial and ethnic health disparities. The Office of Management and Budget (OMB) sets reporting standards for race and ethnicity in Statistical Policy Directive No. 15, a federal policy that guides the minimum categories that must be reported at the national level (3). The minimum OMB race and ethnicity groups included in the standard have not been changed since 1997 (4,5). Questionnaires that follow these standards, including those of the US Census Bureau, have been shown to disproportionately undercount racial and ethnic minority populations (6,7). These groups may have higher rates of missing or unknown responses for the race and ethnicity questions in critical health datasets, because federal agencies such as the US Department of Health and Human Services and state and local health systems design their patient data collection forms in accordance with OMB standards (8,9).

Furthermore, the aggregation of diverse groups into a single racial category obscures within-group disparities and homogenizes people on the basis of unclear, inconsistent definitions of race and ethnicity (9,10). Thus, the federal standard for reporting race and ethnicity in the US perpetuates racism through systemic erasure and invisibility of racial and ethnic minority communities from data, resulting in nonrandom exclusion and suppression of minoritized racial and ethnic groups, which then affects resource allocation and policies.

## Data Disaggregation as a Vehicle for Equity

Collecting and reporting disaggregated race and ethnicity data beyond the OMB minimum standards is widely recommended to address disparities through improved data accuracy and completeness (10,11). However, implementing changes to long-standing data collection practices and collecting more detailed demographic data present a substantial set of challenges. These challenges include mistrust among community members about disclosure of race and ethnicity, institutional buy in, staff knowledge and capacity to collect detailed race and ethnicity data, antiquated data infrastructure that is not able to capture granular categories of race and ethnicity or report these data across data systems and registries (eg, transferring data from patient records to state disease registries), and a lack of resources to support the enhancement and interoperability of data systems (12).

Despite these challenges, health systems that update their data procedures by using evidence-based practices may expect to see improvements in data quality, including higher response rates, and thus complete race and ethnicity data, and fewer patient selections of “not listed,” “prefer not to answer,” “some other race,” or related options for race and ethnicity (13). Such improvements may allow data managers to uncover within-group racial health disparities and support more evidence-based, effective approaches to patient-centered care and other institutional health initiatives (14).

Although data disaggregation research and guidelines have been established by numerous institutions, limited documentation exists on interdisciplinary programs to operationalize evidence-based practices at institutional, local, and state levels. In this essay, we summarize the Innovations in Data Equity for All Laboratory (IDEAL) initiative, a partnership among community-based organizations (CBOs), academic researchers, health care providers, and government officials to transform health data collection in New York State. Below, we broadly describe the activities undertaken by contributors to the initiative to support IDEAL’s overall goal to support community-driven approaches to disaggregating race and ethnicity data in New York State.

## IDEAL Overview and Objectives

A cross-sector, interdisciplinary collaborative, IDEAL aims to 1) strengthen community–academic–government collaborations in discussions of best practices and implementation processes, 2) document and evaluate the process of implementing modified race and ethnicity questions across multiple sectors, 3) update race and ethnicity questionnaires that rely on OMB minimum categories to more accurately capture more granular data at state and city levels, 4) apply innovative statistical methods to improve classification and reclassification of race and ethnicity in existing data, and 5) provide detailed technical assistance on implementation of race and ethnicity data collection, analysis, and reporting procedures.

We will describe activities that IDEAL has initiated thus far to achieve these objectives, all currently in process, with the intention of illustrating potential undertakings for new collaboratives that seek data reform at institutional, local, or regional levels. The activities follow existing recommendations for disaggregating data through equitable, participatory actions, the outcomes of which may be published at a future date.

## Activities

### Strengthening collaborations

IDEAL is led by the Center for the Study of Asian American Health (CSAAH) at New York University (NYU) and the Coalition for Asian American Children and Families (CACF), a long-time CSAAH CBO partner. CSAAH staff members coordinate activities among all contributors to the IDEAL initiative, conduct research to support statistical methodologies, secure funding, and provide other forms of logistical and technical support across teams and activities. CACF leverages partnerships with a coalition of CBOs to reach New York City-wide racial and ethnocultural groups, providing tailored educational materials to explain data disaggregation and its rationale to community and policy makers and leading advocacy activities to ensure accurate data collection and resource allocation. CSAAH and CACF also worked with The New York Academy of Medicine, an IDEAL partner, to recruit diverse participants and facilitate focus groups. 

These efforts were financially supported by the New York State Department of Health, which also convened monthly meetings with CSAAH and CACF throughout 2022 to discuss the applicability of IDEAL key findings to enhance state-level health surveillance data on race and ethnicity. Finally, IDEAL has worked closely with leaders of NYU Langone Health (NYULH) to pilot collection of updated race and ethnicity questions and new data disaggregation practices within the health system.

### Documenting cross-sector implementation

The initiative’s planning stage included researching state-level race and ethnicity data disaggregation efforts. A summary report provided IDEAL with information on model policies, data governance systems, and data collection methods to better understand current efforts to disaggregate race and ethnicity data and other forms of data reformation.

The research scan focused on California, Michigan, and Nevada, states that were previously selected as priority locations by a collaborative of national civil rights organizations to develop strategy plans for enhanced disaggregated data collection, analysis, and reporting. Two other states, Oregon and Minnesota, were also evaluated because their legislatures had passed statutes related to data disaggregation. Narrative summaries of the work in these states provided helpful examples of opportunities and challenges that may arise in similar efforts in New York State.

### Questionnaire development

Self-reported race and ethnicity is a gold standard and preferred source (9); thus, we prioritized community perspectives to guide minimum race and ethnicity categories for inclusion in data collection (eg, patient intake forms). In partnership with CACF and other CBO partners representing diverse racial and ethnocultural communities and The New York Academy of Medicine, we conducted a series of focus groups to gauge community viewpoints on disaggregated race and ethnicity data collection. Participants (N = 81) in 13 virtual focus group and two key informant interviewees, primarily based in New York, responded to questions about their racial and ethnic identity, including how they feel self-identifying in health care settings and how they prefer to identify when completing a questionnaire. Participants also provided feedback on questions about race and ethnicity, including the minimum OMB race and ethnicity categories and proposed race and ethnicity question updates that included more granular options.

Findings indicated that individuals self-identify in diverse ways, even among those who self-identified into OMB-defined racial or ethnic categories. Participants often reported finding difficulty distilling one’s identity into a single closed-ended answer category. Several recommendations emerged, including 1) combining race and ethnicity into one question, 2) limiting the number of answer categories to not overwhelm respondents, 3) allowing multiple responses, and 4) providing information to survey respondents about why race and ethnicity questions are asked and how that information will be used (15).

Analyses of the focus group transcripts, combined with CBO feedback, supported IDEAL’s development of a template questionnaire and recommendations for more inclusive, patient-centered terminology in data collection.

### Statistical methods

Imputing missing information on race and ethnicity in previously collected data can be a helpful step for institutions to begin investigating population demographic characteristics together with disaggregation efforts. The IDEAL team conducted a systematic scoping review of established methods for predicting race and ethnicity in secondary datasets (16). We are currently testing two main methods identified from the review, Bayesian Improved Surname Geocoding and surname list algorithms (17,18). These tests use patient data from the NYULH system and the New York State Immunization Information System to determine whether these two methods are appropriate for imputing missing information on race and ethnicity in our internal datasets for these two patient populations. 

Each of these two methods has unique strengths and limitations and are not appropriate replacements for self-reporting race and ethnicity. However, they can help illuminate racial and ethnic groups that could be incompletely or inaccurately represented in state- and local-level health data.

### Technical assistance

Ongoing community engagement is required for successful dissemination of information about local changes to race and ethnicity data collection. To reach communities, CACF developed handouts to explain data disaggregation in plain language, focusing on the Asian American and Native Hawaiian or Pacific Islander populations, and recently helped pass legislation in New York State that requires state agencies to collect disaggregated data from both of these groups (19). To prepare agencies during the law’s implementation period, IDEAL also developed a comprehensive manual to promote best practices for collecting, analyzing, and reporting health data of Asian American populations (20).

We created toolkits on data disaggregation with explainers, FAQs, and resources designed for a general audience of community members, with separate FAQs tailored for distinct race and ethnicity groups: American Indian/Alaska Native, Asian, Black or African American, Hispanic or Latino, Middle Eastern or North African, Multiracial, Native Hawaiian/Pacific Islander, and White. The primary objectives of these toolkits are to help prepare communities for changes to race and ethnicity language on demographic questionnaires, describe the benefits of disaggregation, and address potential concerns. These documents will be finalized at a future date, and IDEAL will continue to leverage the community partnerships cultivated through this initiative to ensure materials are understandable and adequately address concerns. IDEAL also created toolkits for health care providers (eg, physicians, nurses) and personnel (eg, front desk staff, medical scribes), CBO leaders, and data managers, providing best practices, answers to FAQs, and methodologic recommendations for each of these groups.

### Example of implementation

In 2020, the NYULH began a concerted effort to update its processes for collecting patient data on race and ethnicity. CSAAH worked closely with NYULH leadership, including health care personnel (health care professionals and information technology and administrative staff members), to implement disaggregated data collection practices that are adapted to meet patient and institutional needs. Because NYULH’s data infrastructure was limited by the design flexibility of its electronic medical record software (Epic software), the first implementation step was to design a disaggregated race and ethnicity questionnaire to fit the technologic infrastructure. For example, although branching question logic would improve the experience of data enterers if they were selecting from a lengthy list of race and ethnicity categories, this feature was not enabled in the existing system. Ultimately, the IDEAL team reduced the number of recommended response options, balancing race and ethnicity inclusion with user-friendliness.

As a starting point for disaggregating race and ethnicity data in Epic, CSAAH provided NYULH with its questionnaire template, developed by using the US Census Bureau’s American Community Survey ancestry tables, CBO input, and focus group findings. This process resulted in a list of 67 potential race and ethnicity groups deemed to be the most populous in New York City and relevant to the NYULH patient population. To address feedback about the burdensome number of response options, the IDEAL team narrowed the list from the top 10 to the top 5 ancestries most reported on the 2019 American Community Survey in New York City in each OMB race and ethnicity category, including a category for Middle Eastern or North African and a category for multiracial. Additional groups were added on the basis of recommendations from community and NYULH working partners. The resulting 39 disaggregated race and ethnicity groups allowed the list to fit on a single-page display and prevent patients and health care personnel from becoming overwhelmed by the options at the point of data entry. This updated data collection process provides additional options for category selection among NYULH patients while maintaining the ability to aggregate the more granular groups into OMB minimum standards for federal and state reporting purposes.

Other considerations include developing a plan to ensure NYULH patients and health care professionals and personnel understand the purpose of disaggregated race and ethnicity data collection. A communications strategy encompasses a question-and-answer web page, a postcard mailer, a mass email campaign, and a video series featuring stories of patients and health care personnel demonstrating the importance of race and ethnicity information for health outcomes. Banners at the top of online patient portals will link patients to the question-and-answer page. Lastly, training sessions will be developed for health care administrators and patient-facing staff members, detailing the ethos of disaggregating race and ethnicity data and how to answer patient questions and concerns.

NYULH changes to race and ethnicity data in Epic went into effect on March 9, 2023. The time frame required for adequate understanding of all success factors and barriers to implementation and interoperability is yet to be determined. The IDEAL team will continue working with NYULH leadership and data managers to monitor response rates in all disaggregated categories to continuously make procedural and technical improvements. We anticipate that the perceptions of these changes among patients and health care professionals and personnel will change over time, and we plan to qualitatively assess these experiences during routine discussions with NYULH leaders and health care personnel.

## Next Steps

As the OMB takes its first steps toward revising federal race and ethnicity data standards for the first time since 1997, the IDEAL initiative can be a feasible multisector and collaborative model for other parties seeking to adapt their health data systems, pending changes to the reporting standard and evidence-based best practices (21). The practice of data disaggregation in particular can advance medical data systems in ways that can improve clinical algorithms and serve a culture of patient-centered care.

Our initiative’s racial and ethnic equity- and community-centered approaches to reform medical data strive toward equity at numerous steps of the data pipeline. The methods described, along with ongoing evaluation and quality improvement, can strengthen patient and community trust and are necessary to equitably and effectively document race and ethnicity in health care settings. We will continue to assess these strategies and their impact on data quality and community interests.
